# An ultra-thin double-functional metasurface patch antenna for UHF RFID applications

**DOI:** 10.1038/s41598-020-79506-5

**Published:** 2021-01-13

**Authors:** Mohsen Koohestani, Alireza Ghaneizadeh

**Affiliations:** 1 Department of Electrical and Control Engineering, École Supérieure d’Électronique de l’Ouest (ESEO), 49107 Angers, France; 2 Institut d’Électronique et de Télécommunications de Rennes (IETR), Université de Rennes 1, 35042 Rennes, France; 3 Department of Electrical Engineering, Sadjad University of Technology, Mashhad, 9188148848 Iran

**Keywords:** Electrical and electronic engineering, Surfaces, interfaces and thin films

## Abstract

An ultra-thin double-functional metasurface patch antenna (MPA) was proposed, where it can operate not only in the antenna mode but also can simultaneously act as perfect absorber for normal incident waves, suitable for RFID applications in the 868 MHz band. The MPA structure consists of a typical coaxially-fed patch antenna merged, for the first time, with a metasurface absorber acting as artificial ground. A methodology for the unit-cell design of the metasurface is proposed followed by an equivalent circuit model analysis, which makes it possible to transform a low-loss ($tan\delta =0.0015$) unit-cell with highly-reflective characteristics to a perfect absorber for normal incident waves. It is based on modifying the critical external coupling by properly introducing slits on the unit-cell, allowing to design an ultra-thin ($\lambda _0/225$ at 868 MHz) and a very compact structure in comparison to previously developed designs. For validation purposes, the MPA was fabricated and its performances in both functional modes were characterized numerically and experimentally. It is demonstrated that merging the absorber with the patch not only allows obtaining a well-matched ($|S_{11}|<-30$ dB) antenna with an enhanced gain (by 175.6% compared to a typical patch) at the desired frequency but also leads to an overall thickness of only 2.5 mm ($\lambda _0/138.1$ at 868 MHz). With an absorber size limited to the MPA dimensions, a reasonable 1.3 dB reduction in powers reflected by the MPA was achieved compared to a similar size metallic sheet. Whilst having the lowest profile among the so far reported RFID readers, the proposed MPA can be conveniently fitted for example within the required volume of smart shelf RFID readers or used in portable RFID readers while being capable of mitigating multipath reflection issues and incorrect reading of RFID.

## Introduction

With the current trends in wireless communication systems, low-profile antennas with relatively small vertical cross sections can easily find applications in as diverse as consumer and industrial electronics as well as military and emergency systems^1^. Such antennas are of great demand not only in stationary equipment but also in today’s portable devices which combine wireless communication and navigation with consumer electronics.

Radio frequency identification (RFID) is a well-developed and widely-used technology to wirelessly identify moving and stationary physical objects with seemingly limitless potential for growth. The ultra-high frequency (UHF) RFID band ranging from 865 to 868 MHz in Europe and 902–928 MHz in the US is currently the most popular operating frequency due to offering a good trade-off among the power consumption, read range, security, and penetrating force features in free environment^2^.

The huge drawback of the UHF RFID systems is the severe electromagnetic (EM) interference aggravated with the extent in wavelength. Specifically, in an indoor environment where the communication is strongly influenced by the multiplex reflection interference, the incorrect operation (e.g. in tag read rates and read distances) would deteriorate the reliable communications^3,4^. This fact fostered some interest in the wireless communications community for ways to mitigate the multiplex effect with the aim to provide a correct and precise identification^3–7^.

A most commonly used approach to improve the undesired multipath environment for UHF RFID systems is employing metasurface-based absorbers^3,5–7^. Such absorbers are composition of two-dimensional subwavelength periodic unit-cells, which adapt the incoming wave impedance to that of absorber at resonance frequency^8^. Another prevalent solution is to take benefit from radar absorbing materials such as ferrite or carbon^9^. The former technique is usually preferred as the latter approach is bulky, heavy, and costly. A literature review study reveals that metasurface absorbers are well-investigated mostly in the GHz rather than the MHz frequency range of the UHF band. This stems from the fact that, in the MHz range, such absorbers are rather thick and quite large in size with narrow bandwidth^5,10–13^. Techniques such as employing costly ferrite materials^9^ and using lumped elements in the unit-cell design^14^ can potentially lead to thinner design and increased bandwidth, respectively. As far as the UHF RFID radio link is concerned, the limited bandwidth of such absorbers can be ignored as they require to cover only a few megahertz (i.e. 3 MHz and/or 26 MHz in Europe and in the US, respectively). Therefore, developing thin, compact, and low-cost absorbers would be of great value for the megahertz range of the UHF RFID systems.

The UHF RFID reader patch-based antennas are generally thick, bulky, and heavy, especially the commercially available ones^15–17^. With the aim to possibly cover the entire worldwide UHF RFID bandwidth (860–960 MHz), techniques such as (1) including several air gaps in the antenna design^18–23^, (2) utilizing high dielectric permittivity materials^24^, and (3) using thick substrates^25–27^ were employed. Unlike (1) that enhances the antenna matching bandwidth and gain at the expense of the bulky profile, both (2) and (3) not only lead to narrower antenna bandwidth but also excite more the surface waves in the structure leading to decreased radiation efficiency and directivity. This calls for the design of low-profile antennas with a compromise among the various antenna parameters, provided that the major concern for portable UHF RFID readers is the compactness rather than the antenna gain and bandwidth.

It is generally known that a quarter-wavelength distance is required between the antenna radiating element and the ground plane to mitigate the adverse phase reversal effect of the conductive grounds on the antenna performance^28^. Due to the nature of metasurface structure, if replaced with the antenna ground plane, that distance can be reduced due to the in phase surface currents over the antenna and the artificial ground. Moreover, metasurfaces are capable of stopping the surface wave propagation^28,29^ and can be easily applied to a variety of antenna designs, including patch antennas, which suffer from the adverse effects of surface waves. Metasurfaces integrated with patch antennas, referred to as metasurface patch antennas (MPAs) in the current paper, below the patch acting as artificial ground plane^30–33^ or at the patch level surrounding its circumference^34,35^, have been previously studied mainly in the higher UHF band (i.e. in the GHz range). That combination was found to be effective to enhance the antenna gain^33–35^ and bandwidth^30,32^, to improve front-to-back ratio^32,34^, as well as to suppress the surface wave excitation^28,34^. In the lower UHF band, very few studies have been reported^36–38^. In^36^ and^37^, 300 and 960 MHz dipole antennas were placed above a thick (40 mm) electromagnetic bandgap and a double-layered high-impedance surface (HIS) ground plane, respectively. The HIS structure developed in^37^ led to a gain enhancement of 4.2 dB at 960 MHz. In^38^, a dual-band (915 and 2450 MHz) circularly polarized grounded-patch antenna was located over an HIS; the overall thickness and gain of the antenna at 915 MHz are 30 mm and 3.1 dBi, respectively.

In order to enhance conventional patch antennas performance using metasurfaces, all the so far developed structures were mainly designed using HIS structures due to their great potential to realize low-profile efficient antennas. The interest to employ the absorber rather than the HIS in the MPA structure has not yet been investigated. That combination would provide two different functionality to not only act as an absorber helping mitigate the multiplex reflection interference in the UHF RFID systems but also potentially contribute positively in the antenna characteristics to a certain extent. Therefore, the main goal of this work is to verify the latter aspect demonstrating its pros and cons. To achieve that goal, (1) following a proposed design methodology, a compact ultra-thin metasurface absorber has been designed made of low-cost substrate operating in the megahertz range of the UHF band, and (2) integrated, for the first time, with a coaxially-fed patch antenna in order to build a low-profile RFID reader antenna while assessing how and to what extent that combination would influence the antenna electrical characteristics. Full-wave simulations and measurements have been performed along with comprehensive physical and electrical modeling analyses for the design of the proposed double-functional metasurface patch antenna.

The remainder of the paper is organized as follows. “Geometry of the complete MPA structure” section introduces the geometrical characteristics of the proposed MPA structure. “Analysis and design” section deals with the detailed analysis and design of the proposed metasurface absorber integrated with the coaxially-fed patch antenna. To validate the proposed concept, “Experimental results and discussion” section addresses the analysis of the numerical and experimental results for each functionality of the MPA. Concluding contributions of this study are provided in “Conclusion” section.

## Geometry of the complete MPA structure

**Figure 1:**
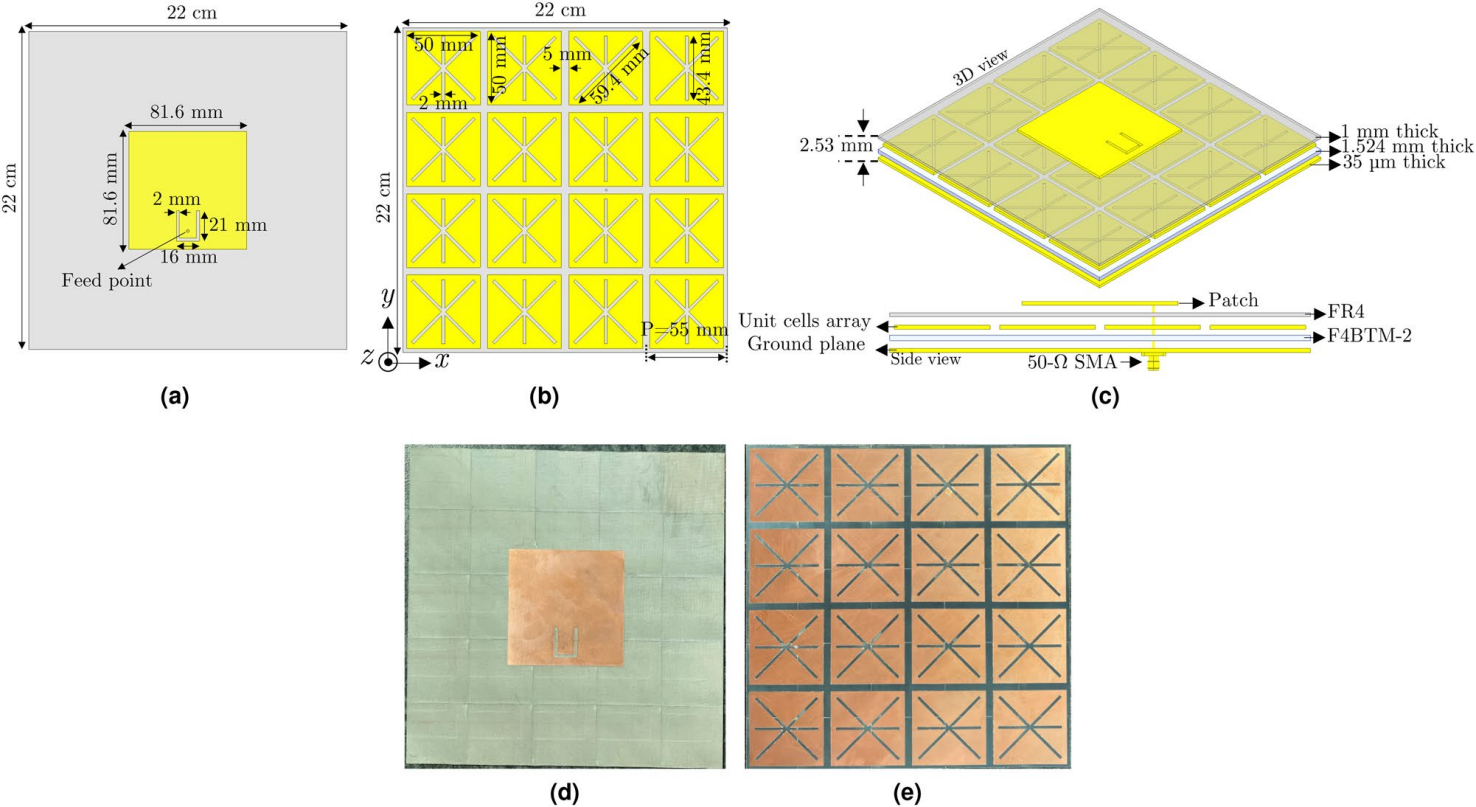
Geometry of the proposed metasurface patch antenna: (**a**) top-view of the patch; (**b**) top-view of the metasurface structure with $4 \times 4$ elements; (**c**) 3D- and side-view of the MPA design; (**d**) top-view of the fabricated patch; (**e**) top-view of the metasurface structure prototype.

The MPA structure proposed in the current study comprises a typical patch antenna (Fig. 1a–d) that its ground plane is substituted with a metasurface absorber structure (Fig. 1b–e). It can be a potential RFID reader as it can operate not only in a normal antenna mode with improved performance but also act as an absorber to suppress scattering, which can effectively reduce the incorrect reading of RFID systems in multipath environment. The geometry and detailed physical parameters of the proposed MPA are provided in Fig. 1c. The patch is printed on a 1-mm-thick inexpensive FR4 epoxy substrate with $\varepsilon _r=4.4$ and $tan\delta =0.02$. The metasurface absorber structure composed of $4\times 4$ unit-cell matrix uses a low-cost and low-loss 1.524-mm-thick F4BTM-2 substrate with $\varepsilon _r=2.55$ and $tan\delta =0.0015$. A laser etching machine (LPKF ProtoLaser S4) was used to realize both the patch and the absorber structures, shown in Fig. 1d,e, respectively. The overall MPA thickness is only 2.53 mm.

## Analysis and design

This section deals with the comprehensive analysis and design of the complete MPA structure consisting of a coaxially-fed patch antenna and a metasurface absorber. The circuit analysis was performed using Keysight ADS software package^39^, whereas the full-wave simulations were carried out with the help of Ansys HFSS electromagnetic solver^40^.

### Coaxially-fed printed patch antenna structure

Seeing that low-profile antennas are in demand, printed patch structures are favored. They, however, suffer from narrow bandwidth and low gain due to the excited surface wave, which traps and dissipates a portion of the radiated energy in the substrate. Thanks to the rapid development of design solutions to overcome those limits, discussed in a precedent section, printed patch antennas are among the most commonly used antenna types.

Since the patch antenna is aimed here to be merged directly with the metasurface structure, due to the existence of the strong mutual coupling effect, the selection of the feeding technique to excite the antenna is crucial. Feeding structures such as microstrip and coplanar lines would lead to destruct the balance of the current distributions on the nearby metasurface unit-cells all along the feeding line connected to the patch. In order to avoid that problem, a coaxial feeding method was considered to excite the patch. Moreover, since linearly-polarized reader antennas typically have longer read range compared to circularly-polarized ones of the same gain, which is due to concentrating the emission in one vertical or horizontal plane rather than across two separated planes, a patch antenna with a linear polarization was chosen for the purpose of the study.

**Figure 2:**
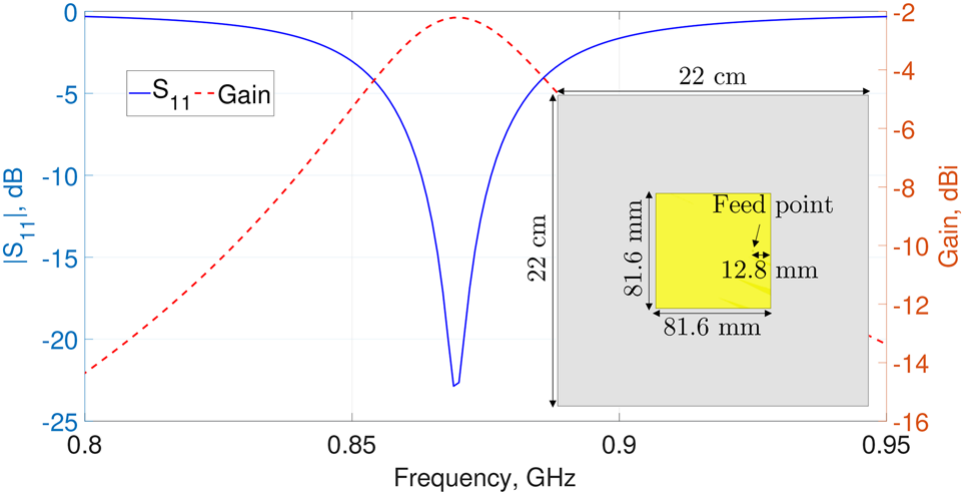
Simulated $|S_{11}|$ and realized gain of a typical patch antenna operating at 868 MHz.

Hence, a typical linearly-polarized patch antenna fed by a coaxial probe was designed to operate in the lower UHF RFID band. Figure 2 depicts the antenna geometry together with its parameters in terms of input reflection coefficient and realized gain. As observed, the antenna has a narrow 10 dB matching bandwidth ranging from 863 to 876 MHz, wide enough to cover the required UHF RFID band in Europe, and a peak realized gain of − 2.25 dBi.

### Metasurface absorber structure

Artificially engineered metasurfaces find extreme applications in radio, microwave and optical frequency regions as diverse as metalenses, high-gain antennas, energy harvesters, transmit/reflectarrays, absorbers, to name a few^41–44^. The basic structure of metasurface absorber consists of an array of unit-cells replicated a number of times in the *x* and *y* directions. The unit-cell comprises three layers: a metal patch, a dielectric, and a conductive ground plane. Here follows a comprehensive analysis for the unit-cell design by proposing a new methodology along with the equivalent circuit modeling.

#### Design methodology

Metasurfaces control the amplitude, phase, and polarization of local fields by manipulating the shape, geometry, and arrangement of the sub-wavelength polarizable inclusions embedded in a host medium^43,44^. The interaction between the incident EM beam and the metasurface absorber can be explained by the induced currents generated from the excited electric and magnetic dipole moments on the unit-cells. A perfect metasurface absorber, assessed by the balance between the internal losses and the external coupling, prevents re-radiation by canceling out the amount of transmission and reflection power coefficinets^29^. Since the backside of the structure is completely covered by copper, transmission coefficient is zero, and hence, the absorptivity (A) can be calculated by A($\omega _0) = 1 - |\Gamma (\omega _0)|^2$, where $\omega _0$ and $\Gamma$ are the angular frequency and reflection coefficient, respectively^41^.

The total reachable absorption is substantially limited to the intrinsic losses of resonant unit-cells. Possible solutions to further improve the absorption characteristics include introducing additional internal losses by resistive materials or effective dielectric losses with appropriate impedance matchings^45^. Another alternative approach is to control the absorption mechanism by adjusting the external coupling rather than the internal losses^46^. The reflection coefficient $\Gamma$ as a function of angular frequency $\omega _0$, internal losses $\frac{1}{\tau _0}$, and external coupling $\frac{1}{\tau _e}$ is expressed as in (1).

1 $$\begin{aligned} \Gamma = \frac{\left( \frac{1}{\tau _{e}}\right) -\left( \frac{1}{\tau _{0}}\right) -j\left( \omega -\omega _{0}\right) }{\left( \frac{1}{\tau _{e}}\right) +\left( \frac{1}{\tau _{0}}\right) +j\left( \omega -\omega _{0}\right) }\Rightarrow \Gamma _{res} = \frac{\left( \frac{1}{\tau _{e}}\right) -\left( \frac{1}{\tau _{0}}\right) }{\left( \frac{1}{\tau _{e}}\right) +\left( \frac{1}{\tau _{0}}\right) }. \end{aligned}$$

For $\frac{1}{\tau _e} =\frac{1}{\tau _0}$, no reflection is experienced at resonant frequency satisfying the condition of critical coupling ($|\Gamma |=0$). The latter can be achieved through a proper design of unit-cells. It should be stated that when $\frac{1}{\tau _e}>\frac{1}{\tau _0}$ and $\frac{1}{\tau _e}<\frac{1}{\tau _0}$, the resonator is so-called to be overcoupled and undercoupled, respectively (see Fig. 7.5 in^46^). In the overcoupled region, the rate of the power escape from the resonating structure (metasurface absorber at resonance) is greater than that of the internal dissipation, i.e. $\frac{1}{\tau _e}~>~\frac{1}{\tau _0}$. For the undercoupled case ($\frac{1}{\tau _e}~<~\frac{1}{\tau _0}$), the amount of internal losses is higher compared to power leakage from the structure. The reflection phase at the resonant frequency is 0$^\circ$ and 180$^\circ$ in the overcoupled and undercoupled regions, respectively.

The above approach was recently implemented in^45^ to design the absorber unit-cell in the millimeter waves (i.e. 30 GHz). However, the drawback is the dependency of the critical coupling condition satisfaction on the overall size of the unit-cell. In the current study, that method was further developed in the megahertz range with the aim to satisfy the critical coupling condition independently of the unit-cell dimensions. We intend to demonstrate that, on the same size and type of substrate, a simple unit-cell with highly-reflective characteristics can be transformed to a perfect absorber for normal incident waves. To achieve a perfect absorption by modifying the external coupling, the latter was controlled by properly creating rectangular slits on the metal patch, which will be explained hereafter.

**Figure 3:**
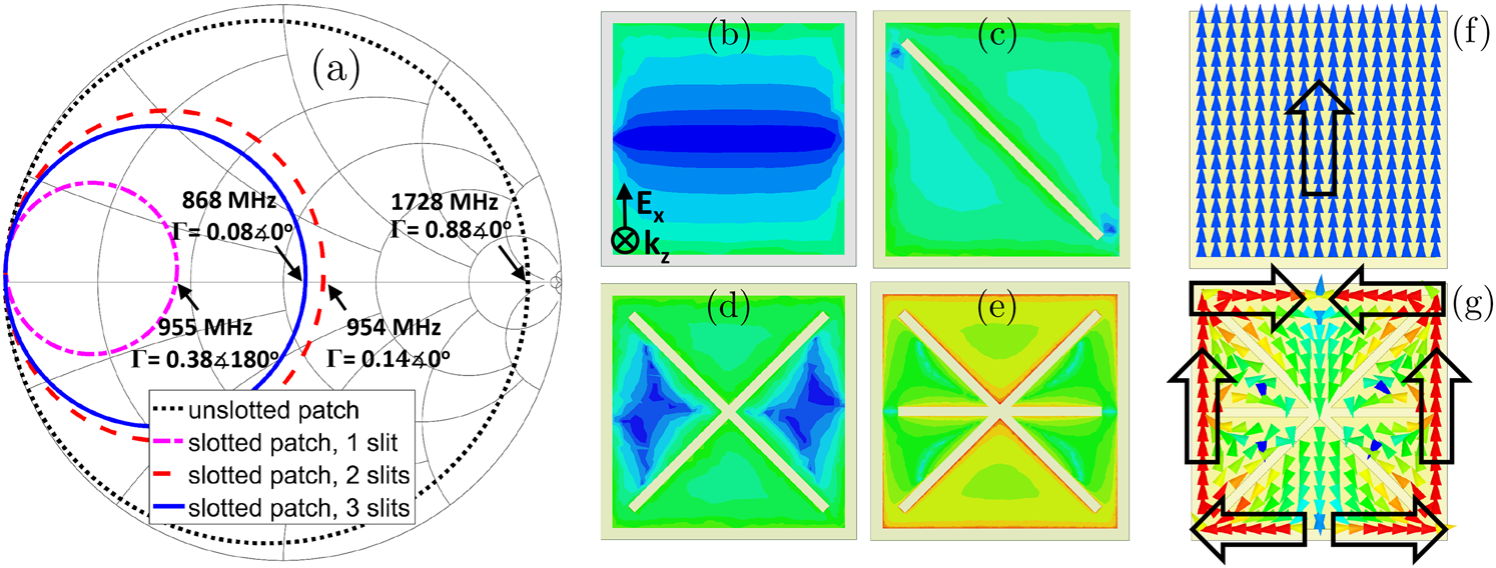
(**a**) Loci of $\Gamma$($\omega$) in Smith-chart for the unit-cell design evolution; E-field distribution on the top surface of the unit-cell design steps for a normal incident planewave at 868 MHz towards the: (**b**) unslotted patch; (**c**)–(**e**) slotted patch with one, two, and three slits, respectively. Vector current distribution on the top surface of the (**f**) unslotted patch and (**g**) proposed unit-cell illuminated by *x*-polarized normal incident wave at 868 MHz. Black arrows indicate the privileged orientation. Scale: logarithmic with 15 subdivisions ranging from 3 to $3\times 10^5$ V/m and 0.1 to 100 A/m.

To assess the unit-cell behavior, a typical set of full-wave simulations were carried out using a normal incident plane wave with periodic boundary conditions parallel to the main axis. The unit-cell design is based on a simple square patch printed on an ultra-thin low-loss substrate with a low dielectric constant. As can be seen in the Smith-chart, shown in Fig. 3a, the unslotted patch lies within the overcoupled region at 1728 MHz ($\Gamma =0.88\measuredangle 0^\circ$) being far from the critical coupling conditions at the frequency of interest. Note that with the E polarization along the *x*-axis (as can be seen in Fig. 3b), the E maximum on the top surface of the unit-cell tends to appear in the edges along the *y*-axis. In order to keep the design compact while achieving acceptable absorption characteristics, slits were etched on the patch to be able to modify the external coupling, due to the power leakage from the structure, until reaching the critical coupling condition.

A first diagonal slit, with the aim to make a longer current path, was created on the patch (Fig. 3c), to shift down the resonant frequency from 1728 to 955 MHz. That also led to move from the overcoupled to undercoupled region ($\Gamma =0.38 \measuredangle 180^\circ$, Fig. 3a). A second slot was introduced to the structure to locally symmetrize the E distribution with respect to the incident wave polarization having its maximum concentrated in the unit-cell edges along the *y*-axis (Fig. 3d). That made it possible to further reach the critical coupling condition ($\Gamma =0.14 \measuredangle 0^\circ$, Fig. 3a) at the same frequency to the case with one slit. In order to reach the desired frequency, an additional slit was introduced horizontally in the middle of the structure where the E distribution is minimal, shown in Fig. 3e, not only to avoid disturbing the E symmetry but also to downshift its resonance frequency by further extending the length of the current path. With an optimized length of the last slit extracted from HFSS, the condition of critical coupling was satisfied at the desired frequency of 868 MHz ($\Gamma =0.08 \measuredangle 0^\circ$, Fig. 3a).

In ultra-thin metasurface absorbers where the perfect absorption is achieved by additional internal losses, the periodicity of the cell (*P*), which greatly affects the absorption frequency, can be approximately calculated as in (2)^41^

2 $$\begin{aligned} P = \frac{\lambda _{m}}{2} = \frac{c}{2f\sqrt{\varepsilon _r}} \end{aligned}$$

where $\lambda _m$, $\varepsilon _r$, *c*, and *f* are the guided wavelength, substrate permittivity, velocity of light, and the resonance frequency, respectively. Although the critical coupling condition to attain perfect absorption was satisfied here by adjusting the external coupling rather than the internal losses, the above formulation can be used provided that the increase of the length of the current path due to the addition of the slits into the unit-cell design is considered. To illustrate the maximum extent in that length, Fig. 3f,g show the vector current distributions on the top surface of the unslotted and proposed unit-cells, respectively. The surface currents are mainly concentrated on the outer edge portions of the proposed unit-cell due to the presence of the slits (Fig. 3g) experiencing a longer path (approximately twice) compared to the unslotted patch (Fig. 3f); the latter resonant frequency is almost twice that of the proposed unit-cell (Fig. 3a). That change in resonant frequency by a factor of two explains the choice of the periodicity value of 55 mm (Fig. 1b) rather than 110 mm (being the approximate *P* value at 868 MHz using (2) for $\varepsilon _r=2.55$). Note that although equation (2) reasonably estimates the resonant frequency of the unslotted patch unit-cell (i.e. 1728 MHz) for $P=55$ mm, due to the absence of any additional internal losses (resistive materials or effective dielectric losses), no absorption should be expected with that unit-cell geometry (as seen in Fig. 3a, |$\Gamma$| = 0.88). The further additions of the slits are indeed required to satisfy the critical external coupling at 868 MHz.

**Figure 4:**
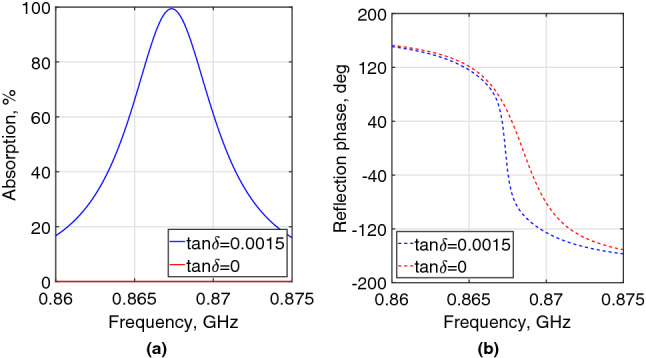
Simulated absorption (**a**) and reflection phase (**b**) of the proposed unit-cell with $tan\delta$ equal to 0.0015 and 0 for a normal incident planewave.

It is worth mentioning that, according to the Poynting theorem, the real part of the average power over time-period is related to the imaginary part of permeability and permittivity^47^. Therefore, the substrate loss tangent is a key parameter to obtain the perfect absorption. The latter was achieved here by the control of the external coupling on a low-loss unit-cell material with $tan\delta$ of only 0.0015. For a better understanding of the loss tangent impact on the absorption characteristics, Fig. 4 depicts the simulated absorption and reflection phase of the proposed unit-cell with $tan\delta$ equal to 0.0015 and 0. As observed, the lossless unit-cell behaves similar to a typical HIS reflector with the unity-magnitude and in-phase reflection (i.e. $+$ 90$^\circ$ to − 90$^\circ$), whereas a nearly perfect absorption was achieved with $tan\delta=0.0015$. The concept and the design procedure introduced in this subsection can be scaled to various frequency bands and generally applied to the design of the absorber unit-cell of any low-loss material.

#### Equivalent circuit analysis

**Figure 5:**
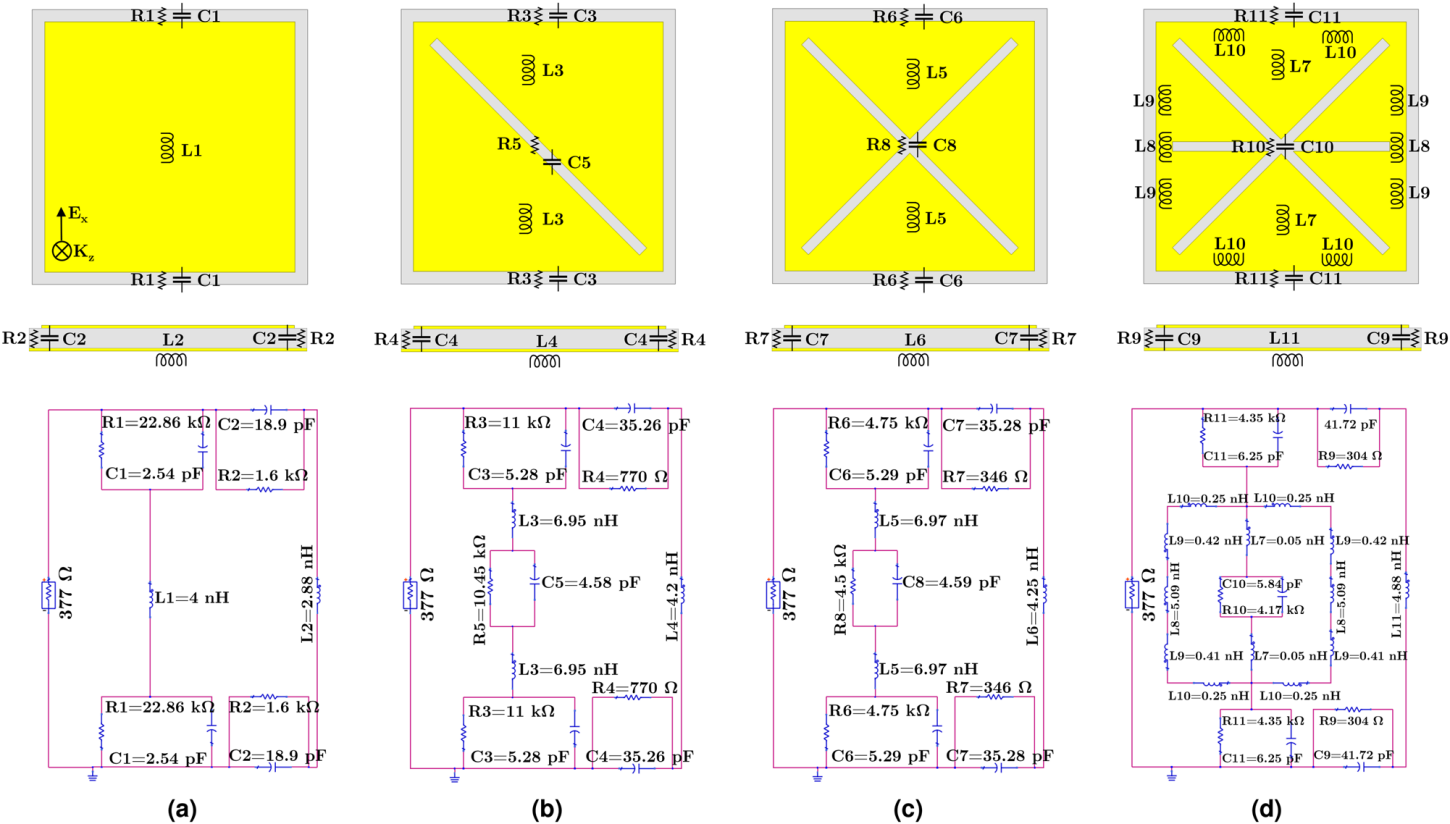
Equivalent electrical model for the unit-cell design steps representing the location of the corresponding lumped components in their structures: (**a**) unslotted patch; (**b**)–(**d**) slotted patch with one, two, and three slits, respectively.

In order to have an insight on the role and effects of the slits introduced in the unit-cell on the absorption mechanism from the circuit point of view, a simplified equivalent circuit model was designed and developed. The unit-cell dielectric can be modeled as a combination of parallel resistor and capacitor representing the dielectric losses and its capacitance, whereas the conductive ground plane can be simply an inductor neglecting the ohmic losses^41^. The unit-cell metal patch can be described using inductors and the addition of the slits calls for extra parallel combination of equivalent resistors and capacitors. For a clearer illustration, Fig. 5 demonstrates the locations of each lumped component on the corresponding unit-cell design steps together with the equivalent electrical model. The input port was set the free space characteristic impedance (i.e. 377 $\Omega$).

A first approximate value was defined for some of the lumped components and the equivalent lumped circuit parameters were then optimized using ADS simulations. For instance, a ratio was considered between the inductors representing the patches based on the surface current intensities extracted from full-wave simulations using current probes defined along the current path at the resonant frequency. Note that additional inductors were introduced in the final model as the surface currents experienced multiple directions along the current path due to the addition of the horizontal slit. The capacitance of the unit-cell substrate was estimated from the microstrip line model. The modeled resistors in the gap area between the unit-cell elements and in the slits were considered to have a higher value compared to those formed between the top and the bottom conductive layers. This is due to the weaker fringing E-field (lower displacement current) within the superficial slots compared to the E-fields existing within the substrate^48^, which further leads to lower capacitance values.

**Figure 6:**
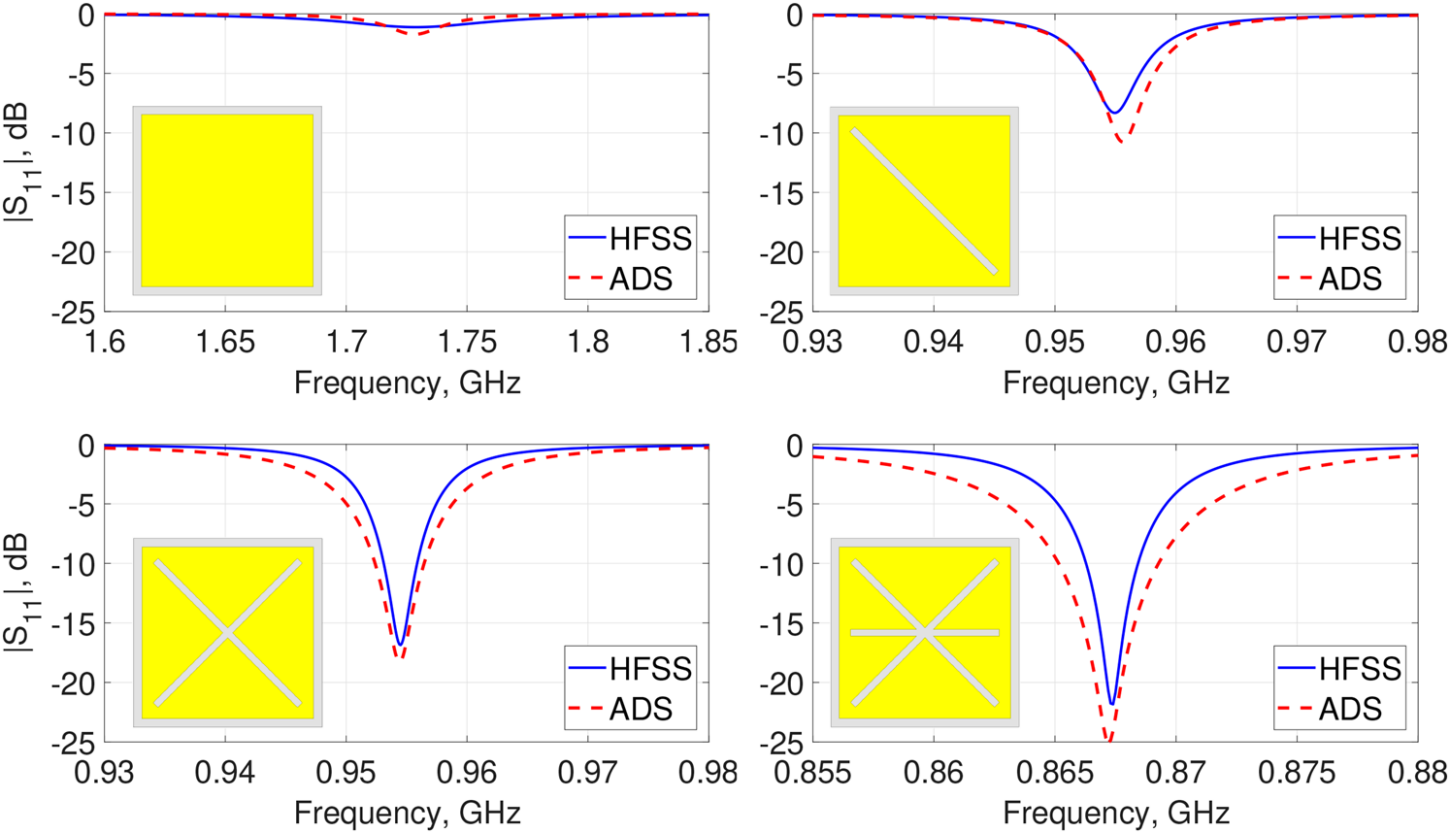
Simulated $|S_{11}|$ results of the unit-cells derived from HFSS and ADS.

Figure 6 compares the $|S_{11}|$ responses for each step of the unit-cell design obtained from ADS and HFSS. As observed, with a reasonable agreement between the results, the circuit model can accurately predict the electromagnetic properties. Moreover, such an equivalent electrical model would further allow manipulate consciously its architecture to achieve the desired performance.

#### State-of-the-art unit-cell designs operating around 868 MHz

Table 1 provides a summary of the so far developed absorbers’ unit-cells with various type patch geometries printed on substrates of different dielectric permittivities and thicknesses. As it can be seen, implementing the proposed design methodology to produce the unit-cell led to an ultra-thin ($\frac{1}{225}\lambda _0$) and a very compact structure when compared to all previously reported designs. Note that although the designs developed in^5,11^ have smaller dimensions compared to our proposed one, their thicknesses are larger by factors of 14.1 and 2.6, respectively.

**Table 1:** Comparison among unit-cells operating around 868 MHz.

References	Unit-cell material	Res. freq. (MHz)	Dimensions $(\hbox {mm}^{\mathrm {3}})$
^3^	Trans. plastic (${\upvarepsilon }_{\mathrm {r}}=$ 3–4, *tan*$\delta \approx$ 0.01)	920	$92 \times 92 \times 5$
^5^	FR4/Air ($\upvarepsilon _{\mathrm {r}}=$ 4.4/1, *tan*$\delta \approx$ 0.02/0)	910	$20 \times 20 \times 21.6, 40 \times 40 \times 21.6$
^6^	PVC plastic ($\upvarepsilon _{\mathrm {r}}=$ 2.68, *tan*$\delta \approx$ 0.01)	920	$100 \times 100 \times 4$
^7^	Cardboard ($\upvarepsilon _{\mathrm {r}}=$ 1.8, *tan*$\delta \approx 0.017)$	868	$162 \times 162 \times 6.5$
^10^	Cardboard ($\upvarepsilon _{\mathrm {r}}=$ 1.45–1.65, *tan*$\delta \approx$ 0.02–0.5)	868	$145 \times 145 \times 8$
^11^	FR4 epoxy ($\upvarepsilon _{\mathrm {r}}=$ 4.4, *tan*$\delta \approx$ 0.02)	900	$45 \times 45 \times 4$
^12^	FR4 epoxy ($\upvarepsilon _{\mathrm {r}}=$ 4.4, *tan*$\delta \approx$ 0.02)	868	$86.3 \times 86.3 \times 3.2$
^13^	FR4 epoxy (${\upvarepsilon }_{\mathrm {r}}=$ 4.2, *tan*$\delta \approx$ 0.02)	878/956	$136.5 \times 136.5 \times 3.2$
This work	F4BTM–2 ($\upvarepsilon _{\mathrm {r}}=$ 2.55, *tan*$\delta \approx$ 0.0015)	868	$55 \times 55 \times 1.524$

It is worth mentioning that the previous designs employed additional internal losses (by resistive materials^10^ or dielectric losses^11^) to achieve a perfect absorption. Thanks to the proposed methodology, the latter was obtained by modifying the external coupling even with a low-loss unit-cell material ($tan\delta =0.0015$). Moreover, its size permits a large number of array elements in a compact area by placing unit-cells in a two-dimensional grid.

### Merging patch antenna and metasurface absorber

Following the goal to not only design an ultra-thin absorber but also to make a low-profile RFID reader antenna, after successful design and introduction of both the patch antenna and the metasurface absorber configurations, they were merged into the complete antenna structure, referred to as MPA (Fig. 1c). This subsection describes in detail the MPA design presenting its electrical characteristics. A comparison was made between the parameters of a typical patch antenna on a conventional ground (Fig. 2) and when placed over the absorber acting as an artificial ground in order to assess the possible improvement brought by the later.

Since the unit-cell of the absorber was analyzed with an incident planewave with the E-field pointing in the *x*-direction, the patch was positioned on the absorber with a similar E polarization along the *x*-axis. The direct combination of the antenna and the absorber is a challenging task due to the strong mutual coupling between them. This would be even more crucial when taking into account the narrow bandwidth of both the patch and the metasurface designs. Therefore, cautions have to be taken about any possible frequency adjustment due to that close proximity.

The impact of the patch antenna substrate on the absorber unit-cell performance was first investigated in HFSS considering a normal incident with periodic boundary conditions. Although the patch leads to disturb the external coupling efficacy on the nearby unit-cells, which can be neglected in a large-scale array, the patch was not taken into account due to not being a repetitive pattern. A tiny downshift of 1.3% was noticed that can be explained as a dielectric loading effect of the antenna substrate on the absorber unit-cell^49^. That frequency shift is expected to be further decreased for a finite structure, it was hence discarded.

**Figure 7:**
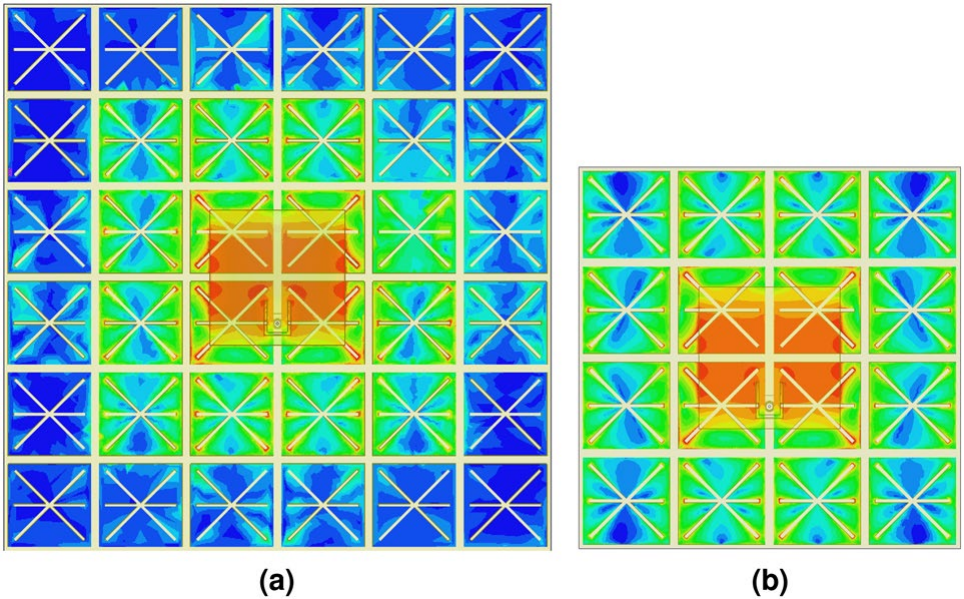
Surface current contributions on the unit-cell array at 868 MHz with (**a**) 6 $\times$ 6 and (**b**) 4 $\times$ 4 element matrix. Scale: logarithmic with 15 subdivisions ranging from 0.06 to 60 A/m.

A metasurface absorber as an artificial ground replacing the conventional patch antenna ground leads to modify the antenna parameters. Before exploring the latter, the number of unit-cell elements, which significantly influences the MPA radiation behavior, has to be determined. A current distribution analysis in a large scale metasurface can provide hints to the minimum number of required unit-cells. Figure 7 depicts the surface current distribution on the unit-cell array of the MPA structure composed of two different element matrices, i.e. 6 $\times$ 6 and 4 $\times$ 4, at the resonant frequency of 868 MHz with 1 W input power injected to the antenna terminal. The colors representing the current in linear scale go from dark blue (weak current density) to green to yellow to red (strong current density). As observed, for the MPA with a $6\times 6$ element matrix, the current is mostly concentrated with a higher intensity over the central $4\times 4$ elements below the radiating patch area (Fig. 7a); an almost similar current distribution can be observed on the unit-cells of the MPA with a 4 $\times$ 4 element matrix (Fig. 7b). That indicates that the size of the absorber area can be reduced to 4 $\times$ 4 unit-cells, while expecting to approximately achieve the maximum radiating properties with the MPA composed of a minimum 4 $\times$ 4 unit-cells in the antenna mode.

**Figure 8:**
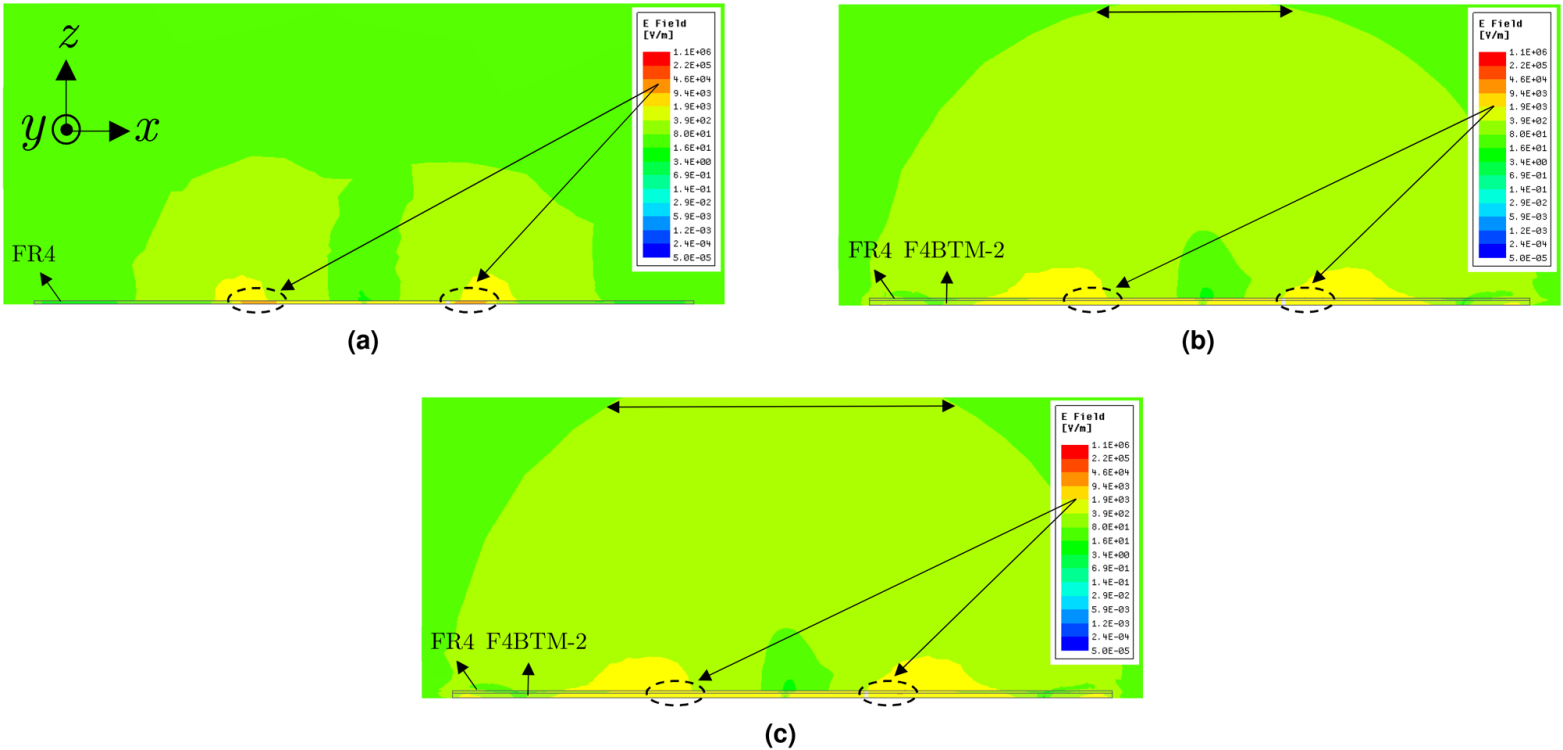
E-field distributions on the *x*–*z* plane for 1 W input power at 868 MHz produced by a: (**a**) typical patch, (**b**) proposed MPA, (**c**) similar to (**b**) with lossless metasurface.

Since the considered artificial ground is a metasurface absorber rather than a high-impedance surface, it is necessary to understand its physical behavior to be able to figure out how it would contribute to the antenna radiating properties. In order to explain that, the loss of the artificial ground (including the substrate and copper layers) in the MPA structure was removed to convert it to a HIS^41,47^ (Fig. 4). The radiated E-field in the lossless case was compared to that of the normal MPA and the typical patch antenna in a defined area in the *x*–*z* plane for an input power of 1 W at 868 MHz (Fig. 8). It can be clearly seen that the outward radiation extends to further distances for the MPA (Fig. 8b) compared to the typical patch (Fig. 8a). This is attributed to the fact that a portion of energy is dissipated in the patch substrate (indicated by dashed-line in Fig. 8) leading to a lower gain compared to the proposed MPA. In other words, replacing the conventional patch ground with the artificial ground, which was designed as an absorber rather than a HIS, contributes positively in the overall antenna radiating properties. However, for the MPA with lossless artificial ground, the extension of the fields into the free space, pointed out by horizontal arrows, is higher (Fig. 8c) compared to that radiated by the normal MPA (Fig. 8b). This means that using the artificial ground designed as absorber contributes slightly negatively to the antenna radiation properties when compared to the ground designed as HIS.

**Figure 9:**
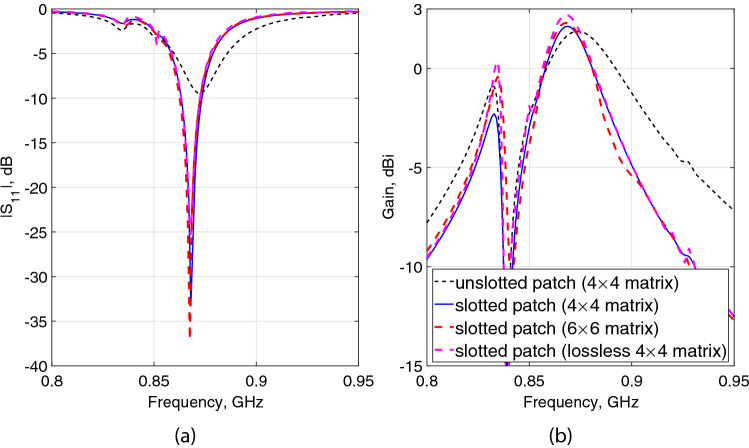
Impact of the unit-cell loss and matrix array on the (**a**) $|S_{11}|$ and (**b**) realized gain of the proposed MPA.

In order to further indicate the extent of that positive and negative contributions to the MPA performance and to verify the effectiveness of the reduced sized absorber when compared to a larger scale one on the MPA radiating characteristics, Fig. 9 shows the impact of the absorber unit-cell’s matrix array and dielectric loss on the $|S_{11}|$ and realized gain of the proposed MPA. The following observations were made:

- the direct integration of the typical patch antenna (Fig. 2) with the final structure of the absorber slightly detuned the patch resonant frequency (shifting up from 868 to 872 MHz, Fig. 9a) and $|S_{11}|$ matching level (increasing from − 22.9 to − 9.5 dB, Fig. 9a). It, however, led to significantly increase the antenna gain by approximately 158.4% (increasing from − 2.25 to 1.87 dBi, Fig. 9b) in line with the observations made and analyzed from the antennas radiated E-fields (Fig. 8). For a fair comparison, to bring the resonant frequency back to 868 MHz and to compensate for the matching level, a small U-shaped slot was etched near the feeding point, labeled as slotted patch in Fig. 9. As it can be seen, the slotted patch (solid-blue) sharply resonates at 868 MHz with a good matching level ($<-$33 dB). In this case, the gain enhances from 1.87 to 2.15 dBi. This means that replacing the conventional ground of the typical patch antenna with the proposed artificial ground leads to significantly increases the gain of about 175.6%.
- reducing the number of absorber unit-cells from $6\times 6$ to $4\times 4$ leads to slightly decrease the gain (from 2.3 to 2.15 dBi, Fig. 9b) with hardly any change in the resonant frequency (Fig. 9a). Therefore, in line with the conclusion made from the surface current analysis (Fig. 7), the size of the absorber area can be indeed reduced to $4\times 4$ unit-cells, while expecting the maximum radiating properties in the antenna mode.
- omitting the loss of the absorber led to a slight increase (from 2.15 to 2.69 dBi) in the MPA gain, as deduced when comparing the E-fields radiated by the normal MPA structure (Fig. 8b) and that by the lossless absorber (Fig. 8c). This can be considered as a moderate cost to use a metasurface absorber instead of an HIS in the MPA design. Note that, in contrast to HIS where the magnitude of the reflection coefficient at the resonant frequency is unity (similar to the PEC/PMC), $|\Gamma |$ can reach approximately zero for a large scale of the proposed absorber (Fig. 3a) following the proposed methodology. Therefore, the combination of the absorber with the typical patch antenna not only leads to notably increase the gain (by 175.6%) but also would potentially help improve the multipath environment of RFID systems by mitigating the severe multiplex reflection interference and collision issues.

**Figure 10:**
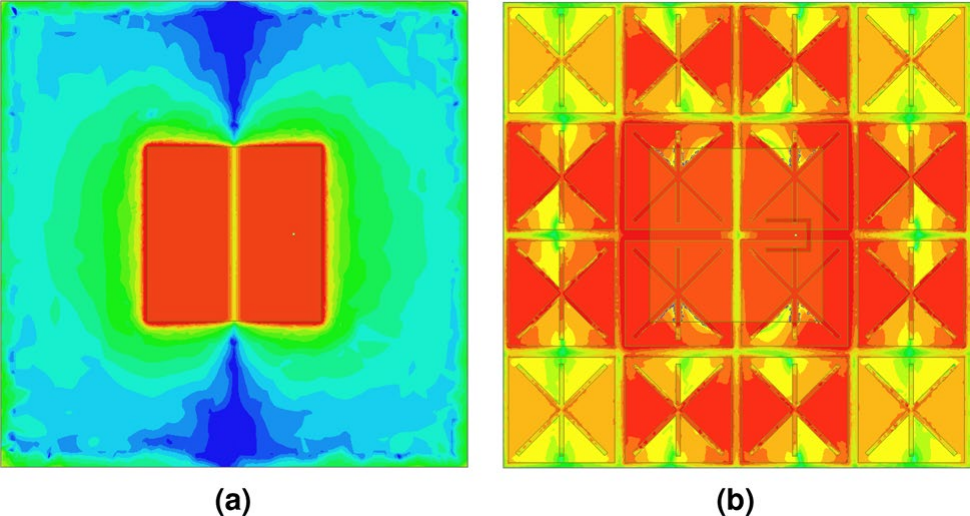
E-field distributions under the FR4 layer on the *x*–*y* plane for 1 W input power at 868 MHz for the (**a**) typical patch antenna; (**b**) MPA structure. Scale: logarithmic with 15 subdivisions ranging from 1 to $2\times 10^3$ V/m.

It should be stated that the enhancement in the gain of the MPA compared to the typical patch can be explained by the increase of the maximum effective aperture of the antenna^50^. Since the physical size of both structures is identical (22 cm $\times$ 22 cm), the artificial ground leads to a higher effective aperture and, consequently, a higher aperture efficiency. The calculated aperture efficiencies for the typical patch and MPA structures are 11.7% and 32.2%, respectively. In order to also visualize that, Fig. 10 depicts the E-field distribution under the FR4 layer of both structures for 1 W input power at 868 MHz. As observed, the maximum effective aperture for the MPA is much larger than that of the typical patch antenna due to the high intensity E distribution all over the entire area (Fig. 10b) rather than only under the patch location (Fig. 10a).

## Experimental results and discussion

To validate the proposed design concept, a prototype was fabricated (Fig. 1). Its performance in terms of input reflection coefficient, gain at the boresight, and radiation pattern was measured in the antenna mode. To have an idea of the absorption characteristics, even though the MPA size is limited, the amount of reflected power from the MPA compared to a metallic plate of similar dimensions was measured in the far-field zone to demonstrate its potential effectiveness in the absorbing mode.

### MPA characteristics in antenna mode

**Figure 11:**
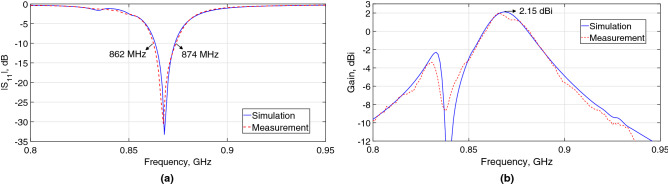
Measured and simulated (**a**) $|S_{11}|$ and (**b**) gain characteristics of the proposed MPA.

The $|S_{11}|$ characteristics of the MPA are shown in Fig. 11a, observing a remarkable agreement between the simulation and measurement results. The − 10 dB $|S_{11}|$ bandwidth fully covers the UHF RFID band in Europe, ranging from 862 to 874 MHz.

The gain characteristics as a function of frequency of the MPA is depicted in Fig. 11b. A reasonable agreement was achieved between the simulated and measured gains. The slight differences between the results can be due to the small drops of the epoxy glue in several areas between the unit-cell gaps, used to stack the patch to the absorber, which was not considered in simulations. The measured maximum gain is 2 dBi in the boresight, closely predicted from the simulation results (2.15 dBi).

**Figure 12:**
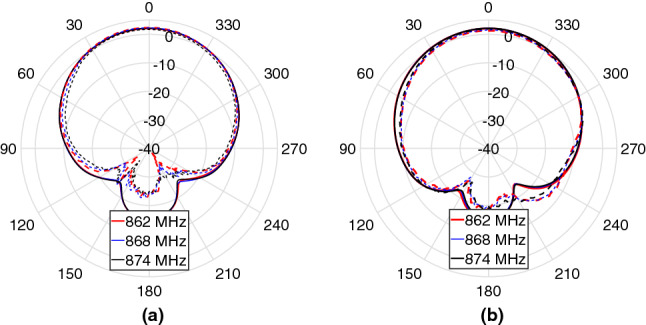
Measured (dashed-line) and simulated (solid-line) radiation patterns of the proposed MPA in the (**a**) *x*–*z* and (**b**) *y*–*z* planes.

The radiation patterns of the MPA at the resonant and 10 dB band edge frequencies in the *x*–*z* and *y*–*z* planes are shown in Fig. 12. A good agreement was obtained between the simulated and measured patterns. The measured (simulated) half-power beamwidth (HPBW) at 868 MHz are 76$^\circ$ (78$^\circ$) and 77$^\circ$ (80$^\circ$) in the *x*–*z* and *y*–*z* planes, respectively. The MPA has a measured front-to-back ratio of approximately 26 dB ($\phi$ = 0$^\circ$) and 21.6 dB ($\phi =90^\circ$) in its entire matching bandwidth. This type of pattern is very well-suited for the target application.

#### State-of-the-art RFID reader designs operating in the UHF band

The obtained results demonstrate the suitability of the approach to combine the metasurface absorber with the typical patch antenna, which results in a low-weight, low-cost, and ultra-thin alternative to current solutions. The proposed MPA dimensions and radiation characteristics are compared to the state-of-the-art RFID reader antennas operating in the UHF band; a summary is provided in Table 2. As it can be seen, not only the commercially available reader antennas^15–17^ but also most of the developed designs in literature^18–23,25^ are bulky and cumbersome, and may not be appropriate for portable RFID applications. They, however, have a higher gain mainly due to using air substrates in their configurations. In^24^, a compact size was achieved using a very high permittivity ceramic material.

**Table 2:** State-of-the-art design of RFID reader antennas operating in the UHF band.

References	BW (MHz)	Gain (dBi)	HPBW	Antenna size (mm$^{\mathrm {3}})$
^15^	902–928	10.5	50°	$305 \times 305 \times 25$
^16^	865–868	7.5	62°	$280 \times 280 \times 12$
	902–928	7.5	62°	$280 \times 280 \times 12$
^17^	865–868	9	54°	$125 \times 125 \times 38$
	902–928	9	54°	$125 \times 125 \times 38$
^18^	840–960	8.3	75°	$250 \times 250 \times 3$5
^19^	855–918	9.3	70°	$200 \times 200 \times 19$
^20^	836–959	8.6	66°	$250 \times 250 \times 36$
^21^	470–790	10.2	$\sim 60^\circ$	$460 \times 460 \times 47$
^22^	838–986	8.6	73°	$250 \times 250 \times 60$
^23^	869–936	5.7	$\sim 80^\circ$	$150 \times 150 \times 12$
^24^	902–928	3	N/A	$40 \times 40 \times 6$
^25^	835–955	7	90°	$200 \times 200 \times 29.8$
^26^	918–929	0.5	100°	$90 \times 90 \times 4.8$
	903–909	3.8	100°	$90 \times 90 \times 4.6$
This work	862–874	2	77°	$220 \times 220 \times 2.5$
	863–877	3.5	92°	$220 \times 220 \times 4.7$
	861–875	1.7	100°	$110 \times 110 \times 4.7$

In^26^, two different compact antenna structures were developed using 4.8-mm-thick FR4 and 4.6-mm-thick RO4003 ($\varepsilon _r=3.38$) substrates. For a fair comparison to that design, in the proposed MPA structure the thickness of the FR4 substrate was first increased from 1 mm to a standard 3.2 mm in order to have an overall thickness comparable to that in^26^. The MPA size was then reduced to a $2\times 2$ array matrix to have a comparable size to that in^26^. Results are given in Table 2. As it can be seen, the proposed MPA provides a higher gain (by 31.8%) and bandwidth (by 27.3%) while having a similar HPBW when compared to that in^26^. It is worth mentioning that for the final MPA size with that increased thickness to 4.7 mm, the gain increased to as high as 3.5 dBi.

To sum up, thanks to its ultra-thin thickness and the degree of freedom in the overall physical size by selecting the number of unit-cell array, with a simple engineering in the structure, similar designs can be created satisfying all the demanding needs with respect to the restrictions imposed depending on the application.

#### MPA characteristics in absorbing mode

The free space measurement method was employed to assess the absorption performance of the MPA structure. In this method, a planewave, in a normal incidence or an oblique angle, is used to excite uniformly the unit-cells of the metasurface absorber^51^. Although a nearly perfect absorption was achieved with the proposed unit-cell (Fig. 4a), a large number of unit-cells has to be considered in the demonstration test sample, where the cells with the largest distance from the center of the structure not only neglect the impact of the central patch presence but also have a minimal influence on the energy absorption^52^. This would not be an issue for the target application provided that RFID readers are often located on the walls, ceilings and/or on the tables, where there would be usually no limit on the physical size. However, due to the limitation to fabricate a large-scale absorber in our laboratory (LPKF ProtoLaser S4 laser machine maximum layout area is 22.9 cm $\times$ 30.5 cm), the prototyped MPA structure including a 4 $\times$ 4 unit-cell array, which was enough to ensure the maximum radiating properties in the antenna mode (Fig. 7), was used for showcasing purposes.

**Figure 13:**
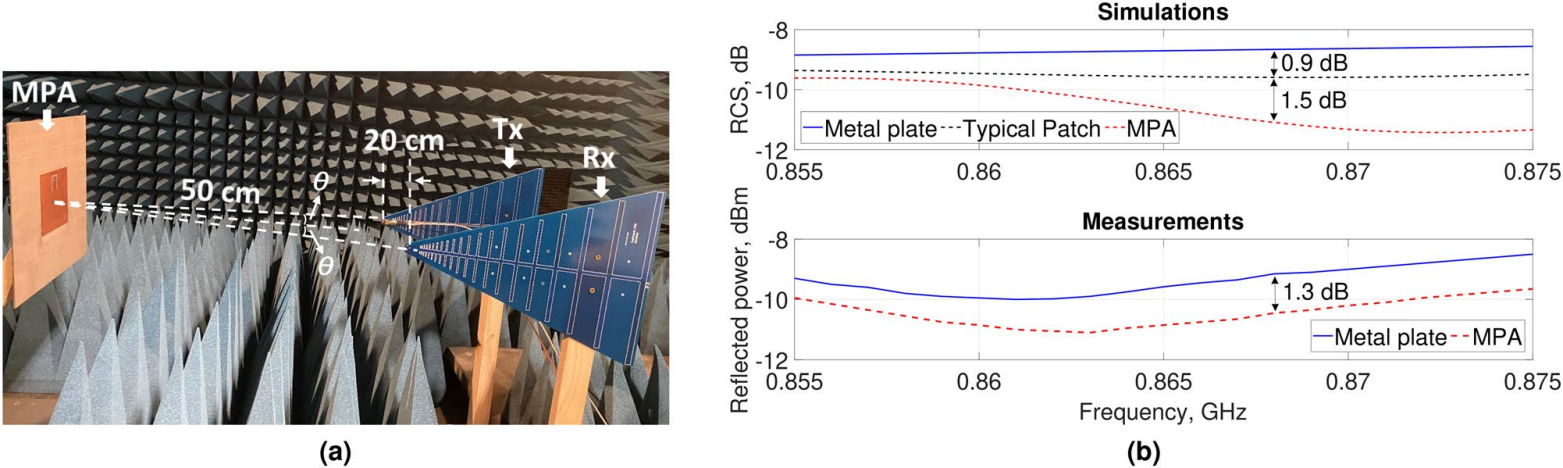
Measurement campaign with the MPA in far-field of two identical log-periodic antennas inside an anechoic chamber: (**a**) experimental setup; (**b**) simulated RCS and measured reflected power.

The measurement campaign inside the anechoic chamber is depicted in Fig. 13a. Two commercially available identical ultra-wideband log-periodic antennas (0.6–16 GHz), one for transmitting (Tx) and one for receiving (Rx), with a 20 cm feed-to-feed distance in between were employed. The input signal generated by an Agilent N9310A (9 kHz–3 GHz) RF signal generator with − 10 dBm input power was amplified by a broadband power amplifier (R&K2737M) with a maximum 28.9 dB gain and further connected to the Tx antenna. The Rx antenna was linked to a Keysight spectrum analyzer (N9320B, 9 kHz–3 GHz). The MPA was placed in the far-field of the log-periodic antennas with a large enough separation distance (50 cm), and then replaced by a same size conducting sheet for a fair comparison to evaluate the absorption performance. In order to have the antennas peak gains coincided with the center of the MPA, both Tx and Rx were positioned by an angle of $\theta =11.5^\circ$; a laser pointer was used to improve the accuracy of the setup.

Since the dimensions of the prototyped absorber is limited to only $0.64\lambda _0$, a perfect absorption around the target frequency of course cannot be expected. In order to have an idea about the absorption characteristics of the structure, a number of full-wave simulations were also performed mimicking the experiments by calculating the radar cross section (RCS) for a planewave with a similar $\theta =11.5^\circ$ oblique incident angle. For a better understanding of the absorption physical mechanism of that limited-size structure, the RCS of a typical patch antenna was also reported. Results are shown in Fig. 13b.

As far as the absorption performance for RFID applications is concerned, in line with the simulations where a 2.4 dB RCS reduction was observed (Fig. 13b), a 1.3 dB decrease in the power levels reflected from the MPA compared to the metal plate was achieved in experiments at the desired frequency (e.g. 0.868 GHz). At frequencies outside the band of interest (e.g. 0.855 GHz), a similar RCS (0.2 dB difference) can be observed for both the typical patch and the MPA, whereas it gradually decreases in the MPA with the increase of the frequency. That reduction reaches its maximum at an up-shifted frequency (e.g. 0.875 GHz), which can be due to the limited number of unit-cell elements in the metasurface structure. A higher reduction can be also noticed in the reflected power from the MPA compared to the metal plate around the frequency band of interest, e.g. at 0.868 GHz compared to 0.855 GHz, both in experiments and simulations. These results demonstrate the potential energy absorption capacity of the reduced-size MPA. An improved absorption performance might be expected for a larger number of unit-cells in the array of the structure, provided that a nearly perfect absorption was obtained with the designed unit-cell element (Fig. 4a).

## Conclusion

An ultra-thin metasurface patch antenna with double functionality (i.e. antenna and absorbing modes) was proposed suitable for RFID applications in the 868 MHz band. The MPA structure comprises a typical coaxially-fed patch antenna merged with a metasurface absorber as artificial ground. The use of metasurface absorber in the antenna structure appeared attractive to design a reader antenna capable of mitigating multipath reflection and incorrect reading of RFID capabilities, which is essential for the considered applications. A design methodology based on adjusting the external coupling rather than the internal losses was proposed to transform a low-loss ($tan\delta =0.0015$) unit-cell with highly-reflective characteristics to a perfect absorber for normal incident waves on the same size and type of substrate. The methodology made it possible to develop an ultra-thin ($\lambda _0/225$ at 868 MHz) and a very compact structure in comparison to previously developed designs. To validate the proposed design, the MPA performance in both functional modes was characterized numerically and experimentally. It was demonstrated that the MPA not only has a 175.6% higher gain when compared to a same size typical patch but also has a low-profile design with overall thickness of only 2.5 mm ($\lambda _0/138.1$ at 868 MHz), which is the lowest profile among the so far reported UHF RFID readers. With the MPA in absorbing mode, taking into account the limit in size, a reasonable reduction of 1.3 dB in powers reflected by the MPA was achieved experimentally compared to a similar size metallic sheet. These results indicate that the proposed MPA is a promising candidate for future portable or stationary RFID applications, competing fairly with so far developed RFID readers.
